# PTSD in Patients Who Undergo Head and Neck Cancer Treatment: A Systematic Review

**DOI:** 10.3390/curroncol32030134

**Published:** 2025-02-26

**Authors:** Orli Weiss, Juliana Runnels, Daniel R. Dickstein, Kristin Hsieh, Lauren Jacobs, Anuja Shah, Danielle Arons, Samuel Reed, Kunal K. Sindhu, Richard Bakst, Julie Bloom

**Affiliations:** Department of Radiation Oncology, Icahn School of Medicine at Mount Sinai, New York, NY 10029, USA; orli.weiss@icahn.mssm.edu (O.W.);

**Keywords:** PTSD, trauma, radiation therapy, head and neck cancer, mental health

## Abstract

Background/Objectives: Post-traumatic stress disorder (PTSD) can develop after exposure to real or perceived threats to life and is characterized by symptoms including intrusive thoughts, hyperarousal, and emotional numbness. While PTSD is well-studied in populations affected by disasters and combat, the impact of serious medical conditions like cancer and its treatments remain under-researched. Due to the aggressive nature of the disease, fear of recurrence, and disfiguring nature of treatments, patients with head and neck cancer (HNC) may experience a real or perceived risk of death. This systematic review synthesizes current knowledge on PTSD in patients with HNC. Methods: A systematic review was conducted per PRISMA guidelines. Five databases (PubMed, EMBASE, SCOPUS, CINAHL, and COCHRANE) were searched for studies describing PTSD in patients with and survivors of HNC. Studies with PTSD diagnosis and/or symptom data specific to patients with HNC were included. Results: Of 80 studies, 14 met the inclusion criteria. The most commonly used scale was the PTSD Checklist-Civilian Version. The prevalence of PTSD ranged from 8% to 41% across the studies. No significant differences were found with regards to PTSD prevalence by HNC tumor site, disease stage, or treatment modality. Two studies identified significant associations between PTSD after treatment and depression at the time of diagnosis. Patients with PTSD who received cognitive behavioral therapy showed improvement in their PTSD symptoms compared to those who did not. Conclusions: PTSD is common in individuals with HNC; however, the lack of a standardized approach to diagnosing PTSD in patients with and survivors of HNC creates challenges in identifying patients who may benefit from treatment. Given that HNC is the seventh most common cancer worldwide, with increasing incidence, there is a need to better understand the relationship between HNC and PTSD to allow for better PTSD screening, identification, and treatment to improve patients’ health-related quality of life and provide optimal patient care.

## 1. Introduction

Head and neck cancer (HNC), as an aggregate is a common, debilitating, and deadly cancer with 890,000 new diagnoses and 450,000 deaths annually worldwide. HNC comprises roughly 4.5% of all new cancer diagnoses and 4.6% of cancer deaths annually [1]. The incidence of HNC is also rising worldwide with increasing consumption of tobacco, alcohol, and areca nut. In more developed nations, the rate of human papillomavirus (HPV)-related cancers, primarily oropharyngeal, is rapidly increasing and is associated with significantly longer median survival [2,3]. Rising mortality numbers appear to reflect the combination of increased incidence with unchanged survival. While there is wide variability across tumor sites, disease stage, etiology, and socioeconomic factors, 5-year survival rate hover around 50% globally [3]. In the US, 27% of cases are classified as localized, 51% are advanced, 15% are metastatic, and 7% are not yet determined at diagnosis [1].

Treatment for HNC is often invasive, multimodal, and complex, typically involving a combination of surgery, chemotherapy, and/or radiation [4]. Furthermore, given the complexity and the proximity of vulnerable organs, management during and after treatment requires multidisciplinary teams outside of medical and radiation oncologists and head and neck surgeons, such as speech and swallow specialists, physical and occupational therapy, registered dietitians, and smoking cessation groups. The thermoplastic masks used to immobilize patients for radiation therapy have been linked to treatment-related anxiety [5,6,7,8]. There are wide ranging acute and late toxicities associated with individual and combined treatment, including mucositis, pain, ototoxicity, dysphagia, feeding tube dependence, dysarthria, dysgeusia, xerostomia, opportunistic infections, aspiration, and osteonecrosis [4,9,10,11,12,13,14]. These side effects, especially xerostomia and swallowing deficits, have been shown to negatively impact health-related quality of life (HRQOL), as well as physical, mental, and social health [15]. Vital aspects of enjoying life (e.g., deriving pleasure from favorite foods) and maintaining interpersonal relationships (e.g., holding a conversation with friends or loved ones) may be severely compromised. Studies investigating HRQOL in this population have demonstrated mixed results, though even 10 years after treatment there has been evidence of persistent impairment [16,17,18,19]. Anxiety, advanced disease, difficulty swallowing, and feelings of sadness are associated with decisional regret [20,21].

The morbidity of the HNC-directed treatments can have significant impact on mental health [22]. Squamous cell carcinoma (SCC) of the head and neck preferentially affects men, older patients, and those of low socioeconomic status. These groups may be susceptible to post-traumatic stress disorder (PTSD), or they may lack social support and resources to cope with their diagnosis and treatments [1]. Of all cancer patients, those with HNC have the highest anxiety and depression scores after treatment. They have also been observed to experience the most distress, highest suicide rate, and least social functioning [23,24,25,26]. Post-traumatic stress symptoms (PTSS) and anxiety are common in both HNC patients and their partners [27]. Moschopoulo et al. assessed 93 HNC survivors six years after treatment, with approximately one-third exhibiting PTSS and 11.8% meeting the criteria for PTSD [28]. A meta-analysis of mental health in HNC examined 208 studies (n = 654,413) and identified significant rates of patient-reported distress 34.3%, PTSS 17.7%, depression 19.5%, anxiety 17.8%, and insomnia 43.8% [29]. This is in contrast to diagnostic criteria assessments, which yielded lower rates of disorders, for example: PTSS 1%, depression 10.3%, anxiety 5.6%, insomnia 9.6%. Of the 208 studies included, only 3 studies (n = 180) studied PTSS, with an overall prevalence of 0.010 [29]. Furthermore, another study on 817 patients with HNC yielded significantly higher scores across all domains, including depression, anxiety, fatigue, QOL, and psychosocial distress, compared to the general population [30].

The aim of this review article is to critically evaluate the literature on PTSD in the HNC population. Head and neck cancer patients are uniquely affected by a combination of disease factors and treatment-related morbidity, including deficits in basic functioning and visible disfigurement. It is important to understand the state of our current knowledge in order to guide future research and better prevent and manage potential negative impacts on HNC patients’ mental health.

## 2. Methods

A systematic review was conducted as per Preferred Reporting Items for Systematic Reviews and Meta-Analyses (PRISMA) guidelines [31]. The study protocol was registered with the OSF Generalized Systematic Review Registration platform (Registration DOI: https://doi.org/10.17605/OSF.IO/D9W7X; Registration date: 5 February 2025).

This review was conducted in October 2024 through a systematic search of five databases, PubMed (MEDLINE), EMBASE, CINAHL, SCOPUS, and Cochrane, from inception through 9 October 2024. The following search algorithm was used: (ptsd OR “post-traumatic stress disorder” OR emotional trauma OR “post-traumatic stress disorder”) AND (“head and neck cancer” OR “Head and Neck Neoplasm” OR “Cancer of Head and Neck” OR “Head cancer” OR “Neck cancer” OR “neck neoplasm” OR “head neoplasm”). Keywords included post-traumatic stress disorder, trauma, and head and neck cancer.

We included randomized control trials, focus group studies, cohort studies, cross sectional studies, and review articles describing adult patients with HNC diagnoses that provided specific information regarding PTSD, post-traumatic stress, and acute stress disorder symptoms for HNC patients specifically. Exclusion criteria were: (i) studies that presented PTSD data for several types of cancers including non-HNC diagnoses without differentiating between PTSD data for each individual cancer type; (ii) studies that presented PTSD in caregivers only without providing patient data as well; (iii) case reports and letters to the editor; (iv) studies without an available English-language translation; and (v) registered experimental protocols and study overviews without available results.

Search results were uploaded into Covidence screening software [https://app.covidence.org; accessed on 17 October 2024], and duplicates were automatically removed. Two reviewers (O.W. and J.R.) independently screened titles and abstracts using Covidence software, and discrepancies on initial screen were resolved via a third independent reviewer (J.B.). This screening method was repeated for full text review. Data extraction was manually performed for included studies by two reviewers (O.W. and D.D.), with publication year, article title, country, study aims, study design, population description, total number of participants, total number of participants with HNC, PTSD score used, other scores used, and relevant data recorded in a shared spreadsheet file. Given the broad range of study types included in this review, results were grouped and discussed according to study type, location performed, sample size, PTSD prevalence, PTSD scales used, and identified risk factors. Risk of bias assessment was performed for all included studies using the Critical Appraisal Skills Programme Checklists [32].

## 3. Results

### 3.1. Study Design and Study Characteristics

**Figure 1:**
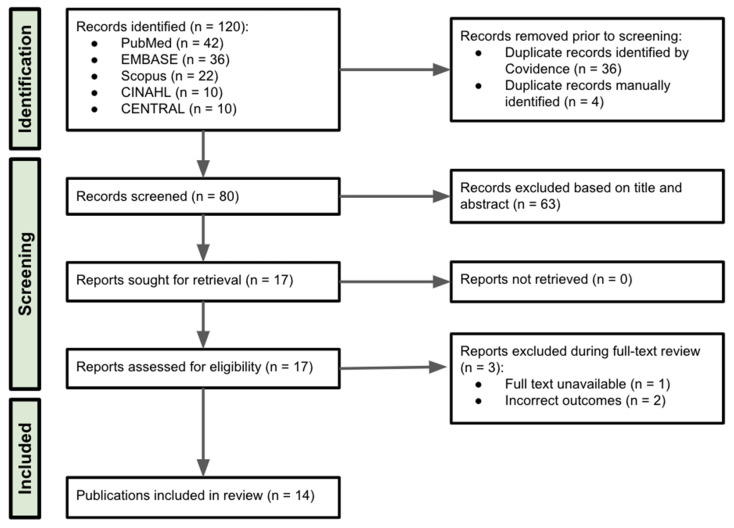
Preferred Reporting Items for Systematic Reviews and Meta-Analyses (PRISMA) Flow Diagram.

From the 80 articles identified through five databases (Figure 1), 63 were excluded during the title and abstract screening. Of the 17 full text articles reviewed, 3 were excluded due to incorrect outcome (n = 2) [33,34] or lack of full text (n = 1) [35] (Figure 1). Fourteen studies were included for final analysis (Table 1) [27,28,29,36,37,38,39,40,41,42,43,44,45,46]: seven cross-sectional studies [27,28,37,38,41,42,45], three cohort studies [36,39,40], two meta-analyses [29,46], one qualitative study [43], and one randomized controlled trial [44]. These studies were conducted across eight countries: the United States (n = 4) [27,36,37,42], United Kingdom (n = 2) [28,41], Australia (n = 1) [44], Canada (n = 1) [43], Germany (n = 1) [38], Japan (n = 1) [45], New Zealand (n = 1) [40], and Spain (n = 1) [39]. Two meta-analyses were conducted by UK-based institutions [29,46], with one involving Spanish collaboration [29]. The studies were published between 2012 and 2024.

See Table 1 for a full description of included studies. 

Sample sizes varied widely. Studies focusing on multiple cancer types [36,37,38,39] included up to 5289 patients [38], with head and neck cancer subgroups ranging from 17 [39] to 278 participants [38]. Head and neck cancer patients comprised 6.5% [36], 40% [37], 5.3% [38], and 26% [39] of these study cohorts. Single-site studies on head and neck cancer patients [27,28,40,41,42,43,44,45] had sample sizes ranging from 29 [43] to 124 [41] participants, averaging 61 patients. Three studies also included caregivers or partners [27,28,42].

PTSD data was collected at specified time points in seven studies [27,28,37,39,40,44,45]. Five studies focused on newly diagnosed cancer patients [27,39,40,44,45]. Three of these studies followed patients over the course of six months to one year following the diagnosis [39,40,44]. One study analyzed patient’s PTSD symptoms at six months following diagnosis only [37], and one study focused on patients who had completed HNC treatment and were at least two years from the diagnosis [28].

### 3.2. Assessment of PTSD Prevalence

Included studies defined PTSD according to the Diagnostic and Statistical Manual of Mental Health Disorders diagnostic criteria of symptoms of intrusive thoughts, avoidant behaviors, negative symptoms, and hyperarousal relating to experience of a traumatic event and lasting more than 30 days following exposure to the trauma [47]. Included studies either referenced PTSD directly (n = 13) [27,28,29,36,37,38,39,40,41,42,44,45,46] or included patient-provided quotations describing PTSD symptoms (n = 1) [43].

PTSD prevalence rates varied across studies. Rates of PTSD ranged from approximately 8% [38] to 41% [45]. The 11 included quantitative studies utilized several validated tools to assess the prevalence of PTSD and related symptoms [27,28,36,37,38,39,40,41,42,44,45]. Three studies utilized the PTSD Checklist-Civilian Version (PCL-C), which uses DSM-IV diagnostic criteria to provide a numeric score for each PTSD symptom cluster (hyperarousal, intrusive thoughts, and avoidance) [27,28,39]. Two studies utilized the Impact of Event Scale-Revised (IES-R), which measures distress symptoms relating to past experience of a traumatic event in the domains of hyperarousal, intrusion, and avoidance [42,45]. Two studies utilized the Primary Care PTSD Screen (PC-PTSD), which provides four yes-no questions intended to evaluate PTSD symptoms of intrusion, avoidance, hyperarousal, and negative symptoms including emotional numbness [36,37]. Two studies utilized the Post-traumatic Stress Disorder Checklist-Stressor Specific Version (PCL-S), where questions to evaluate PTSD symptoms were specifically amended to refer to cancer [37,44]. One study utilized the Clinician Administered PTSD Scale (CAPS), a clinical interview which assesses PTSD symptoms related to cancer using DSM-IV diagnostic criteria [44]. One study utilized the PTSD Scale Self Report (PSS-SR), which assesses PTSD symptoms and their severity using DSM-III-R diagnostic criteria [40]. One study utilized the Acute Stress Disorder Scale (ASD Scale), a scale assessing acute stress disorder symptoms with a score cutoff for probable PTSD caseness [41]. One study utilized the ICD-10 diagnostic codes [38].

### 3.3. PTSD Symptoms by Symptom Cluster

Four studies provided further quantitative analysis of PTSD symptoms by symptom cluster (hyperarousal, intrusive thoughts, avoidance) in patients with HNC [27,28,42,45]. Posluszny et al. reported that patients most often met PTSD criteria for symptoms of intrusion (38.1%), followed by hyperarousal (23.8%), and avoidance (16.7%) [27]. Moschopoulou et al. found similar results, with intrusion being the most common (33.3%), followed by hyperarousal (29%), and avoidance (20.4%) in HNC patients overall. In a subset of HNC patients whose partners also completed PTSD assessments, intrusion was again the most common (35.9%), followed by hyperarousal (33.3%), and avoidance (25.6%) [28]. Yamaguchi et al. focused exclusively on patients with oral cancer and found the highest median IES-R scores for intrusion, followed closely by avoidance, and then hyperarousal [45]. Wang et al. focused on patients with HNC related tracheostomy, and found these patients had highest mean IES-R scores for avoidance, followed by intrusion, and then hyperarousal [42].

### 3.4. Treatment Modality and Number of Treatments Received

Three studies compared PTSD scores based on treatment modality specifically for patients with HNC [27,28,40]. Both Posluszny et al. and Moschopoulou et al. compared PCL-C scores between patients receiving surgery alone, surgery and radiation therapy, surgery and chemoradiation, or chemoradiation, with Moschopoulou additionally analyzing radiation therapy alone and other treatments [27,28]. Both studies found no significant difference in PCL-C score based on treatment modality(ies) [27,28]. Richardson et al. compared patients receiving surgery with patients receiving radiotherapy, and found no significant difference between patients receiving different treatment modalities [40].

Two studies analyzed PTSD scores based on the number of treatments received [40,42]. Wang et al. found that the number of treatment modalities (radiation therapy, chemotherapy, and/or immunotherapy in combination with surgery) patients received was weakly correlated with both physical (r(21) = 0.475, *p* = 0.026) and psychological symptoms (r(21) = 0.438, *p* = 0.041), though specific data corresponding to which type of treatment patients received in addition to surgery was not available [42]. Richardson found no significant difference between patients receiving single modality treatment versus multimodal treatment [40].

### 3.5. Stage and Site

Four studies assessed whether disease stage impacts PTSD scoring in patients with HNC specifically, finding no difference in post-traumatic stress in patients with different disease stages [27,28,40,45]. Although no significant difference in PTSD symptoms was found between patients with different disease stages, one study found quality of life to be significantly higher for patients with earlier disease stages than later stages [40]. Two studies assessed whether disease site impacts PTSD scoring in patients with HNC specifically, finding no differences in post-traumatic stress between HNC sites [28,45].

### 3.6. Clinical Outcomes

Three studies included results related to disfigurement due to HNC treatment [27,28,43]. Two studies provided quantitative data, with conflicting results [27,28]. Posluszny et al. found no relationship between disfigurement and PCL-C scores [27], while Moschopoulou et al. found significant association of PCL-C scores with appearance concerns [28]. One study provided qualitative emotional responses regarding negative emotional responses to disfigurement and treatment related speech impairments which may contribute to avoidant behaviors. [43] One study analyzes the prevalence of PTSD symptoms specifically in patients who received HNC related tracheostomies, finding correlation between physical and psychological symptoms related to tracheostomy and global distress [42].

### 3.7. Risk Factors

Several studies identified risk factors for PTSD in head and neck cancer patients. Moschopoulou et al. found that a previous diagnosis of depression and anxiety was correlated with a higher risk of distress symptoms and PTSD, but did not find statistical significance for this effect [28]. Posluszny et al. found significant positive correlation between patients’ Hospital Anxiety and Depression Scores and PCL-C scores (r = 0.76, *p* = 0.001 for anxiety; r = 0.42, *p* = 0.006 for depression) [27]. Kangas et al. found that, of 14 patients meeting PTSD criteria at baseline, all had either comorbid or subclinical major depressive disorder [44]. Two studies examined the correlation between quality of life (QOL) and PTSD, finding an inverse correlation between QOL and distress symptoms and acute stress disorder [41] and a similar negative correlation between QOL and PCL-C scores [28]. Richardson et al. studied coping strategies used by patients as predictors of PTSD symptoms, finding denial, substance use, behavioral disengagement, venting, humor, and self-blame were also predictors of higher PTSD scores six months post-diagnosis [40]. In contrast, most studies found no significant associations between PTSD and demographic or clinical factors such as age [27,40,41,42,45], gender [40,41,42,45], employment status [45], income level [42], or marital status [45], with one study finding increased PTSS in patients of younger age [28].

### 3.8. Preventative Therapies

One study compared PTSD symptoms in patients newly diagnosed with HNC who received either cognitive behavioral therapy (CBT) or supportive counseling to determine whether preemptive CBT offered additional advantages for reducing acute cancer related PTSD development [44]. Kangas et al. found both interventions statistically equivalent in their ability to reduce PTSD symptoms over time, but found a higher proportion of patients who received CBT had improvement in their PTSD symptoms when compared to the supportive counseling group [44].

### 3.9. PTSD in Partners and Caregivers of Patients with HNC

Three studies also evaluated PTSD and related symptoms in partners [27,28] or family caregivers of patients with HNC [42]. Studies analyzing patients and their partners found that patients’ PTSD symptom scores did not correlate to those of their partners [27,28], both in newly diagnosed patients [27], and for post-treatment patients at least two years from initial diagnosis [28]. Moschopoulou et al. found no significant difference between PTSD prevalence in patients and their partners two years following diagnosis [28]. Posluszny et al. found significantly higher PTSD symptom levels in partners than in patients within 4–16 weeks following diagnosis [27]. Wang et al. found that PTSD levels were similar in caregivers and patients with HNC related tracheostomies, but did not provide information regarding the correlation between patient and caregiver scores [42].

## 4. Discussion

We present the first systematic review dedicated to PTSD in patients with HNC. The small number of included studies reflects the limited reported data on PTSD in patients with HNC and highlights the previously scant interest in and understanding of the importance and nuances of this topic.

There is a dearth of literature on the effects of various trauma, including physical, emotional, or psychological trauma, on the utilization of cancer-related services for patients with cancers on seeking or undergoing cancer-related services. In a 2023 review, Marshall et al. summarizes the effects of trauma on the utilization of cancer-related services, including screening, diagnosis, and management, for patients with cancers [48]. The authors specifically excluded trauma secondary to cancer diagnosis or related care. Correspondingly, there is limited literature on PTSD among patients with cancer, particularly where PTSD secondary to cancer-related care is not excluded. There are two such reviews included in our study, specifically Swartzman et al. assessing the prevalence of PTSD after cancer diagnosis [46] and Jimenez-Labaig et al. reporting the prevalence of post-traumatic symptoms in patients with HNC though there is insufficient data for additional analysis [29]. In contrast, our manuscript comprehensively assesses PTSD symptoms prior to, during, and after cancer-directed therapy and accounts for cancer as a traumatic stressor.

The 2023 review by Marshall et al. reports the level of distress during cancer-related care, compliance with cancer screening and other cancer-related care, and distress management in patients with cancer as the assessed outcome variables [48]. The available literature on the relationship between PTSD and cancer-related outcomes, such as cancer progression-free survival and overall survival, is limited and this relationship is not reported in any of the included studies in the review of Marshall et al. and our review. Thus, correlating PTSD in patients with HNC and their cancer-related outcomes is worthy of additional research. Moreover, the increasing awareness and recognition of PTSD in patients with cancers have led to an emphasis on the creation and implementation of practice guidelines for trauma-informed care that may improve patient satisfaction, adherence to care, and outcomes [49].

There is a wide range in incidence of reported PTSD and PTSD symptoms within the included studies in this systematic review. Prevalence of PTSD ranges from as little as 8% to 41% in some studies [38,45]; the mean occurrence of those studies that reported incidence was 17.6% (IQR: 12,19) [27,28,38,40,44,45]. In studies that reported subclinical PTSD or patients experiencing one cluster symptoms of PTSD, the incidence significantly rose, over tripling, from 46% to 57% [28,44]. The range in incidence may be due to several factors including variability in the assessment tools used and the populations included. One study required elevated levels of anxiety, depression or PTSD prior to enrolling in the pilot study [44]; however, this study, similar to others, reported an incidence of 14% PTSD at baseline. Additionally, patients self-selecting for enrollment in these studies versus exploring databases with inclusion of all HNC patients may be more indicative of the general population without the bias for self selection [38,44].

Head and neck cancer treatments can include a combination of surgery, ranging from minimally invasive surgery to extensive open surgery involving free-flaps and tissue grafts, radiotherapy, ranging from photon radiotherapy to proton therapy, and systemic therapy [4]. Patients receive one or more treatment modalities based on disease site, stage, and characteristics. These treatments differ in their invasiveness, treatment duration, and side effects caused, including pain [4]. Of 14 included studies describing PTSD in HNC patients, three studies compared PTSD in HNC patients receiving different therapeutic modalities [27,28,40], with no study finding significant association between treatment modality and PTSD prevalence; however, the smaller sample sizes (n = 93 [28], n = 42 [27], n = 65 [40]) with even lower numbers of patients receiving each therapy modality may limit current ability to uncover significant associations. Two included studies assessed the impact of number of treatment modalities on PTSD symptoms, with one study finding no association [40] and the other finding a weak positive correlation between number of therapy modalities and both physical and psychological symptoms of distress in patients with HNC related tracheostomy [42]. While different treatment modalities may contribute unique risk factors for PTSD, the current literature may not provide sufficient data to assess PTSD prevalence based on treatment received.

Radiation therapy presents unique risk factors for psychological distress for HNC patients. To receive radiation therapy, patients are immobilized using a thermoplastic mask covering the face, neck, and shoulders. Immobilization minimizes patient movement, allowing for targeted radiation dosing of desired areas and decreased dosing of surrounding healthy structures; however, many patients report claustrophobia and anxiety associated with mask immobilization [5,6,7,8]. Additionally, side effects of radiation therapy can be distressing, including early side effects of xerostomia, dysgeusia, dysphagia, mucositis, and pain [9,10,13,14]. During and shortly after treatment, patients may become unable to eat due to pain, requiring interventions including gastric tube placement for nutrition [9,10,13,14]. Long term side effects can include permanent damage to salivary glands, persistent dysgeusia, osteonecrosis, dental caries, and perioral tissue fibrosis [11,12,13,14]. These side effects can contribute to a significant psychosocial burden for patients [11,15]. Furthermore, radiation therapy requires daily treatments for several weeks, meaning patients are exposed to repeated stresses over the course of treatment.

Providers use several tools to mitigate risks of developing PTSD in patients receiving radiotherapy for HNC. Several centers utilize open face masks for radiotherapy, where patients’ faces are left uncovered, which can ease claustrophobia and anxiety [50]. Additional studies support use of augmented reality training for patients prior to radiation therapy to enhance familiarity with the radiation machines and treatment setup before patients experience immobilization and treatment firsthand [51]. Focused interviews with patients regarding anxiety associated with HNC treatment found allowing patients to choose music during radiation therapy and supportive communication from radiation therapists and physicians to be helpful [7,8]. Additionally, anxiolytic medications can be helpful for some patients [7].

This review has several limitations. Firstly, this manuscript is limited by the quality and quantity of the reported data from the included studies, as well as the methodology and analytical design in those studies. As there was high variability in the methodology of the included studies, especially within the scales used to measure PTSD and related symptoms, we were unable to perform a meta-analysis with the available data. Furthermore, given that the majority of the included articles are based in Europe or North America, our findings may not be representative of patients with HNC worldwide, particularly those with diverse sociocultural backgrounds. Altogether, these limitations may affect the conclusions drawn from our review.

## 5. Future Directions

Future investigations are warranted in this unique patient population for both identification and management of patients with HNC who receive cancer-directed therapy. From this systematic review, we identified that patients with pre-existing depression show correlation with development of PTSD [28]. An abstract published by Pequeno et al. found pre-existing depression to be significantly associated with the development of PTSD in patients with HNC [35]. As clinicians already gather data regarding past medical history, perhaps early intervention with cognitive behavioral therapy and/or medication could reduce the psychological consequences of cancer-directed therapy. Patients who received CBT may be less likely to experience PTSD, anxiety and/or depression at 12 months compared to those who only received supportive counseling [44]. Further exploration of other conditions, including but not limited to denial, substance use, and behavioral tendencies such as disengagement and self-blame should be explored. Furthermore, it would be beneficial to standardize assessment tools for assessing patients undergoing head and neck cancer targeted therapy in order to provide more patient-centered care.

## 6. Conclusions

This systematic review represents the first dedicated effort to synthesize the current literature on PTSD in patients with head and neck cancer, emphasizing its significance as a critical yet underexplored area of research. The limited number of included studies and the variability in methodologies underscore the need for more robust and standardized research to better characterize PTSD prevalence and outcomes in this population. Our results demonstrate that a significant portion of people with HNC who undergo treatment experience PTSD following their diagnosis and treatment. The unique psychological stressors associated with HNC and its treatments highlight the importance of tailored, trauma-informed interventions to mitigate PTSD risk and address its impact early in a patient’s care. Evidence suggests that pre-existing depression is a key risk factor, warranting early intervention strategies, such as cognitive behavioral therapy, to improve patient outcomes. Future research should prioritize the development of standardized assessment tools and evidence-based interventions to address the nuanced needs of HNC patients, while also exploring the relationship between PTSD and cancer-related outcomes. By advancing this area of study, we can improve the identification, prevention, and management of PTSD, ultimately enhancing the quality of life and care for people with head and neck cancer.

## Figures and Tables

**Table 1:** Characteristics of studies included in this systematic review.

Study ID	Title	Study Design	Study Population	Total *n*	*n* with HNC	PTSD Assessment Tool(s)	Results
Costa-Requena 2014 [39]	A One-Year Follow-up of Post-Traumatic Stress Disorder (PTSD) Symptoms and Perceived Social Support in Cancer	Cohort	Patients with HNC, breast and colorectal cancer	66	17 (26%)	PCL-C	-PTSD symptoms showed differences based on time of assessment: mean PCL-C score for HNC patients was highest at the end of treatment (31.33), 29.53 pre-treatment, and lowest 6 months post treatment (27.45). Symptoms of PTSD rose again at 1 year post treatment (30.35).
Henry 2016 [43]	Recovering function and surviving treatments are primary motivators for health behavior change in patients with head and neck cancer: Qualitative focus group study	Focus group	Patients with HNC	29	29 (100%)	*-*	-Focus groups taped, transcribed, and analyzed to understand barriers to and facilitators of health behavior change -9 focus groups with 2–5 participants each. Qualitative data with quotations showing PTSD symptoms -“Other emotional aspects were related to avoidance of the unpleasant experiences associated with the various transition periods, such as the fear of using the PEG tube or food aversions associated with the pain of radiotherapy or the trauma of PEG insertion/removal, and feeling embarrassed and discouraged socially by such treatment side effects as disfigurement, eating in public with the PEG tube, or having a speech impairment” -“Disengagement was related to intense treatment side effects (especially pain and fatigue), emotional aspects (e.g., despair, trauma, discouragement, grief), lack of information, and factors related to the medical team, such as lack of continuity of care, missed referrals to team members, or inadequate pain management”.
Jimenez-Labaig 2024 [29]	A comprehensive examination of mental health in patients with head and neck cancer: systematic review and meta-analysis	Systematic review and meta- analysis	Patients with HNC	654,413	654,413 (100%)	PCL-C PSS-SR IES-R	-Meta-analysis of 208 studies; 3 described Post-traumatic symptoms (n = 180); 3 included studies described PTSD assessment (n = 344) -Prevalence of post-traumatic symptoms: 0.177 (95% CI = 0.061–0.413); prevalence of PTSD: 0.010 (95% CI = 0.000–0.848)
Kangas 2013 [44]	A pilot randomized controlled trial of a brief early intervention for reducing post-traumatic stress disorder, anxiety and depressive symptoms in newly diagnosed head and neck cancer patients	RCT	Patients with HNC	35	35 (100%)	PCL-S CAPS	-HNC patients with elevated levels of PTSD, depression or anxiety undergoing radiotherapy randomized to early CBT program or non-directive supportive counseling. -At baseline, 14% of these patients meet PTSD diagnostic criteria; all of these patients had comorbid or sub-clinical MDD; 57% of patients met the criteria for subclinical PTSD; 26% met the criteria for adjustment disorder. -CBT and supportive counseling both reduced PTSD and anxiety in the short and long term; a larger proportion of patients who received CBT (67%) compared to supportive counseling (25%) no longer met clinical or subclinical PTSD, anxiety or depression at 1 year
Moschopoulou 2018 [28]	Post-traumatic stress in head and neck cancer survivors and their partners	Cross sectional	Patients with HNC, post-treatment and 2 or more years from diagnosis, and their partners	132	93 (70%)	PCL-C	-Analysis of 93 patients with HNC and 39 patient-partner pairs -11.8% of patients met the criteria for PTSD; 46.3% met the criteria for at least one PTSD symptom cluster. 33.3% met the criteria for intrusion, 29% for hyperarousal and 20.4% for avoidance -Subgroup analysis of patients whose partners participated in the analysis revealed that 15.4% met the criteria for PTSD and 15.4% met the criteria for estimated PTSD caseness. 48.7% met the criteria for at least one PTSD symptom cluster. -Patients and partners did not have significantly different PTSS scores, but these scores did not correlate
Nipp 2018 [36]	Symptoms of post-traumatic stress disorder among hospitalized patients with cancer	Cohort	Patients with multiple cancer types	954	62 (6.5%)	PC-PTSD	-Linear regression did not find significant correlation between HNC diagnosis and PTSD cases (95% CI, −0.16–0.46; *p* = 0.350)
Posluszny 2015 [27]	Post-traumatic stress disorder symptoms in newly diagnosed patients with head and neck cancer and their partners	Cross sectional	Patients with HNC and their partners	42	42 (100%)	PCL-C	-Analysis performed on surveys from n = 42 patient-partner dyads -Partners had a significantly higher PTSD symptom burden than patients (28.6% of partners vs. 11.9% of patients, *p* = 0.023) -Cluster based analysis found both patients and partners endorse intrusion symptoms most commonly. -There was no significant relationship between age, disease-related general blame, substance-related blame, disease stage, whether the patient received surgery, amount of disfigurement, whether the patients were currently receiving treatment, and the time since treatment began and patients’ PCL-C scores.
Richardson 2016 [40]	Coping strategies predict post-traumatic stress in patients with head and neck cancer	Cohort	Patients with HNC	65	65 (100%)	PSS-SR	-19% of patients had PSS-SR scores meeting PTSD criteria at 6 months follow-up -There was no significant correlation between age, stage, treatment modality (surgery vs. radiotherapy), receiving 1 vs. combined treatment modalities and post-traumatic stress or HRQL -Coping strategies including denial, substance use, behavioral disengagement, venting, and self blame were associated with higher post-traumatic stress and lower HRQL. Humor was associated with higher post-traumatic stress.
Salm 2021 [38]	Mental disorders and utilization of mental health services in newly diagnosed cancer patients: An analysis of German health insurance claims data	Cross sectional	Patients with multiple cancer types	5289	278 (5%)	ICD-10 codes	-Insurance databases used to obtain results -Of HNC patients, 7.91% contained PTSD/adjustment disorder codes -77.27% of patients with HNC and PTSD/AD utilized psychotherapy and 54.55% utilized pharmacotherapy
Shiraz 2014 [41]	Quality of life, psychological wellbeing, and treatment needs of trauma and head and neck cancer patients	Cross sectional	Patients with HNC and trauma patients	220	124 (56%)	ASD scale	-37% of HNC patients exhibited high scores for depression, 44% high scores for anxiety, and 12% high scores for acute stress disorder. -Quality of Life had an inverse correlation with scores for depression (z = −3.39, *p* < 0.05), anxiety (z = −3.02, *p* < 0.05), and acute stress (z = −3.39, *p* < 0.05) in patients with HNC. -No significant differences due to age or sex.
Wang 2022 [42]	Post-Traumatic Distress and Symptom Experience in Patients With Head and Neck Cancer-Related Tracheostomy and Family Caregivers	Cross sectional	Patients with HNC and tracheostomy, their caregivers	39	22 (56%)	IES-R	-Analysis performed to assess tracheostomy related post-traumatic stress -7/22 patients, 5/17 caregivers had IES-R scores reflecting probable PTSD -Patients had highest mean symptom scores for avoidance, followed by intrusion, and hyperarousal -No significant correlation between post-traumatic distress and age, gender, ethnicity, education, or annual income -Number of treatment modalities received was associated with a significant weak positive correlation with both physical (*p* = 0.026) and psychological (*p* = 0.041) symptoms.
Yamaguchi 2022 [45]	Psychological impact on patients with oral cancer before undergoing resection and free flap reconstruction surgery	Cross sectional	Patients with HNC	34	34 (100%)	IES-R	-Pre-treatment PTSD screen for patients diagnosed with oral cancer and scheduled for surgical resection and free flap reconstruction -41.2% of patients experienced PTSD symptoms -No statistically significant differences in scores by age, gender, primary site, cancer stage, employment, and marital status, or drinking and smoking status in the past year
Swartzman 2017 [46]	Post-traumatic stress disorder after cancer diagnosis in adults: A meta-analysis	Systematic review and meta-analysis	Patients with multiple cancer types	-	-	Multiple	-One study described patients with HNC; proportion of patients with PTSD was 0.3 (95% CI 0.0–2.3)
Wachen 2014 [37]	Cancer-related PTSD symptoms in a veteran sample: association with age, combat PTSD, and quality of life	Cross sectional	Patients with multiple cancer types	166	67 (40%)	PCL-S	-Sample of military veterans -Mean PCL-S score for head and neck cancer patients 30.39; of all the cancers, most patients (86%) experienced at least some cancer-related PTSS, 10% scored above clinical cutoff for probable PTSD

Abbreviations: AD—Adjustment disorder; ASD Scale—Acute Stress Disorder Scale; CAPS—Clinician Administered PTSD Scale; CBT—cognitive behavioral therapy; HNC—head and neck cancer; HRQL: health related quality of life; IES-R—Impact of Event Scale—Revised; ICD-10—International Classification of Diseases, Tenth Revision; MDD—major depressive disorder; PCL-C—PTSD Checklist-Civilian Version; PCL-S—Post-traumatic Stress Disorder Checklist—Stressor Specific Version; PSS-SR—PTSD Scale Self Report; PTSD—post-traumatic stress disorder; PC-PTSD—Primary Care PTSD Screen; RCT—Randomized controlled trial.
